# On the convective heat and zero nanoparticle mass flux conditions in the flow of 3D MHD Couple Stress nanofluid over an exponentially stretched surface

**DOI:** 10.1038/s41598-018-37267-2

**Published:** 2019-01-24

**Authors:** Muhammad Ramzan, Mohsen Sheikholeslami, Maria Saeed, Jae Dong Chung

**Affiliations:** 1 Department of Computer Science, Bahria University, Islamabad Campus, Islamabad, 44000 Pakistan; 2 Department of Mechanical Engineering, Sejong University, Seoul, 143-747 Korea; 3 Department of Mechanical Engineering, Babol Noshirvani University of Technology, Babol, Iran; 4 Department of Mathematics, Allama Iqbal Open University, Islamabad, 44000 Pakistan

## Abstract

Three dimensional problems reflect more imperative understanding to real world issues in comparison to two dimensional problems. Keeping this fact in mind, a mathematical model is designed to deliberate the 3D magnetohydrodynamic couple stress nanofluid flow with joule heating and viscous dissipation effects past an exponential stretched surface. The analysis is performed keeping in mind the physical effects of Brownian motion and thermophoresis combined with convective heat condition. This paper also distinctly introduces a more realistic boundary constraint for nanoliquid flow model. For instance, zero mass flux condition has been instituted for the first time for 3D couple stress nanofluid model as far as the exponential stretched surface is concerned. Self-similar transformations are engaged to obtain a system of ordinary differential equations possessing high nonlinearity from the system of boundary layer partial differential equations. Analytic solution is constructed in the form of series using Homotopy Analysis Method (HAM). Numerically calculated values of Skin friction and local Nusselt number are also given with suitable analysis. Moreover, the influences of sundry parameters on velocity distribution, and heat and mass transfer rates are deliberated and depicted through relevant graphs. The results obtained clearly show that the Biot number and Hartmann number possess increasing effect on temperature distribution. To authenticate our obtained results, a comparison in limiting case is also given.

## Introduction

The term “nanofluid” refers to the nanoparticles (having size less than 100 nm) suspended into the base fluid. Typical examples of nanoparticles include metals such as Copper, Aluminum and Silver, oxides e.g., Aluminum Oxide, carbides such as Silicon Carbides, nitrides like Silicon Nitride and Aluminum Nitride, and nonmetals such as graphite or carbon nanotubes. The customary fluids are ethylene glycol, water, and oil. The amalgamation of nanoparticles with the common fluid tremendously improves the thermal traits of the base fluid.

Choi and Eastman^1^ were the pioneer to introduce the term nanofluid and the fact that several heat transfer physiognomies of the base fluids, such as thermal conductivity is enhanced by insertion of nanoparticles into it. Later, Wang and Arun^2^ deliberated that convective characteristics of base fluid are enhanced by addition of metallic and non-metallic particles into it. This was followed by an experimental study by Eastman^3^ who claimed that thermal conductivity of the ethylene glycol is improved by 40% once copper nanoparticles are inserted in it. Subsequently, Eastman^4^ also examined that the shape of nanoparticles has a pivotal role in increasing the thermal conductivity of base fluid. The decree of Eastman was verified by Murshed^5^ who studied that the amalgamation of water with spherical shaped nanoparticles (Titanium oxide) with sizes more than 40 nm, increases the thermal conductivity of the base fluid by 33%. The use of nanofluids is very common in cooling the transformers and nuclear reactors. In medical, magneto nanofluids are also being utilized in cancer treatment, hyperthermia and MRI (Magnetic Resonance Imaging). The use of nanofluids is also imperative in making the germs free surgical instruments and removal of tumors.

In the literature, generally nanofluid flow can be modelled in two ways. Since the nanoparticles are smaller in size and can be mixed effortlessly in the base fluid so in the first case nanofluid is considered as a single phase flow^6^. In this case the dispersion of the nanoparticles in the base fluid is uniform and stable. Here, the impact of nanoparticles may be taken into consideration by deliberating the thermophysical characteristics of the nanofluids in the model equations. In the second case, named as two-phase flow, the association between liquid matrix and the nanoparticles is considered^7^. The two phase model was introduced by Buongiorno^8^, however, Tiwari and Das^9^ initiated the single phase model. Following these two proposed models, numerous researchers deliberated the thermal role of nanofluids to analyze the effective fluid characteristics^10–19^.

Investigation of non-Newtonian fluids is still a subject of curiosity for scientists and researchers because of their numerous applications in engineering and industry. Examples of non-Newtonian fluids may embrace shampoo, ketchup, polymer solutions, paper pulp and paints etc. Because of many complexities, non-Newtonian fluids cannot be expressed by a solitary constitutive relation in contrast to Newtonian fluids^20^. Therefore, several mathematical models for studying non-Newtonian fluids have been suggested by researchers in the past.

Couple stress fluid model is one amongst various proposed viscoelastic fluid models that exhibits behavior of the non-Newtonian fluids. Couple stress fluid model is considered as generalization of classical fluid model (*i*.*e*., the viscous fluid), and is comprises of couple stresses and body couples^21^. Examples of couple stress fluids may include animal and human blood, colloidal fluids, liquid crystals and liquids with long chain molecules. Basically, in these kind of fluids, the constitutive equations associate angular part of the velocity to the gradient of the angular velocity and the stress tensor’s skew symmetric part to the couple stress^22^. Erigen^23^ was the pioneer who used the term micropolar fluid for polar fluid; whereas dipolar fluids are recognized by their initiators Bleustein and Green^24^. The juncture of polar and dipolar fluids is phrased as couple stress fluids and was introduced by Stokes^25^. As the couple stress fluid’s stress tensor is not symmetric therefore Navier-Stokes equations are not adequate to model such fluids. Thus, the fluids with solid particles dangling in a viscid medium *i*.*e*., the synthetic fluids, the lubricants with small amount of polymer preservative and the blood may be treated as couple stress fluids^26^.

On account of such important applications of couple stress fluid in various engineering fields, numerous researchers and authors have highlighted the various aspects of such fluids. Amongst these, Ramzan *et al*.^27^ found analytical solution via Homotopy analysis method (HAM) of three-dimensional couple stress fluid flow with Newtonian heating. Khan *et al*.^28^ examined numerical solution of time dependent magneto hydrodynamic couple stress fluid flow past a rotating disk. Hayat *et al*.^29^ deliberated analytic solution of three-dimensional magneto hydrodynamic couple stress nanofluid flow past a nonlinear stretched surface with convective heat and mass boundary conditions. Ramzan^30^ found series solutions of three-dimensional couple stress nanofluid flow with joule heating using HAM. Lately, Hayat *et al*.^31^ investigated series solution of three-dimensional magneto hydrodynamic couple stress nanofluid flow with heat generation/absorption under the effects of convective condition. Recently, Hayat *et al*.^32^ deliberated the 3D couple stress nanofluid flow in attendance of Cattaneo- Christov heat flux.

From aforementioned literature review, it is found that flow of 3D magneto hydrodynamic couple stress nanofluid past an exponential stretched surface with convective heat and zero mass flux conditions is still a scarce. Effects of viscous dissipation and Joule heating are also deliberated to analyze of the flow problem. The system of partial differential equations acquired from boundary layer theory are converted to set of nonlinear differential equations using apposite transformations. Renowned Homotopy analysis method (HAM)^33–41^ is engaged to solve this system of ordinary differential equations. Graphical images depicting impacts of varied parameters on involved distributions with mandatory conversation are also given. An appraisal of the presented results to a previous study is also given to authenticate our results.

## Mathematical Modelling

**Figure 1:**
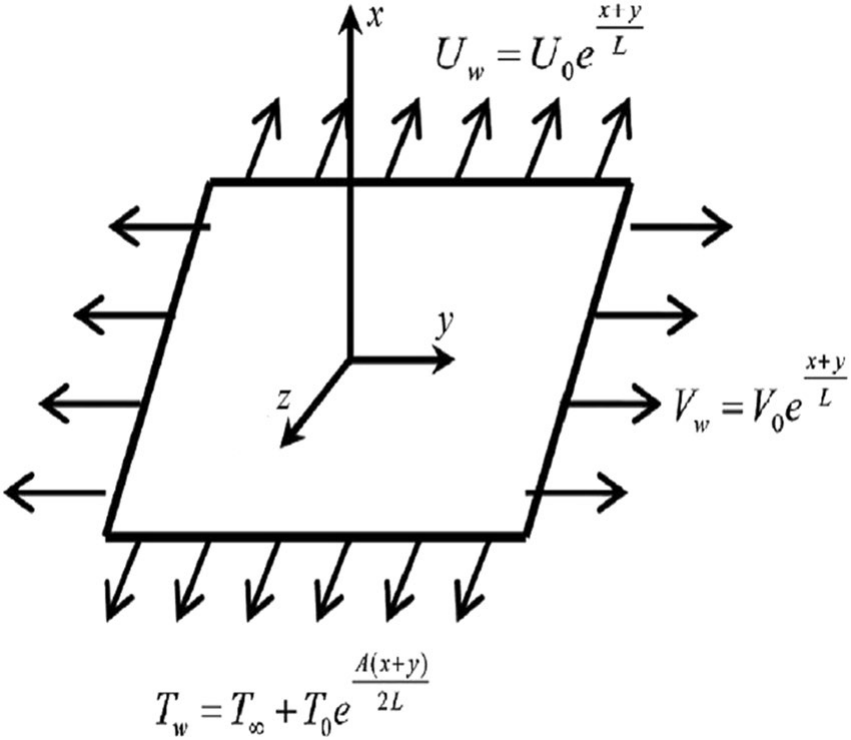
Fluid flow geometry.

Consider a 3D incompressible couple stress nanofluid flow past an exponential stretched surface with convective heat and zero mass flux conditions. It is assumed that $u={U}_{w}(x,y)={U}_{0}{e}^{\tfrac{x+y}{L}},$ and $\upsilon ={V}_{w}(x,y)={V}_{0}{e}^{\tfrac{x+y}{L}}$(with *U*_0_, *V*_0_ are constants) are stretched velocities of the surface along *x*− and *y*− directions respectively. Impact of Joule heating and viscous dissipation are also considered. Couple stress nanofluid is electrically conducting with uniform magnetic field in a direction along *z*− axis (Fig. 1).

It is presumed that magnetic Reynolds number is small and owing to this assumption, the induced magnetic field is ignored when we compare it with the applied magnetic field. Also, (*u*, *v*, *w*) are velocities components along (*x*, *y*, *z*) directions respectively. Also, *T*, *C*, *T*_∞_ and *C*_∞_ represent the fluid’s temperature, the concentration, the ambient temperature and the ambient concentration respectively. Fluid flow is represented by the boundary layer equations as appended below:

1 $$\frac{\partial u}{\partial x}+\frac{\partial \upsilon }{\partial y}+\frac{\partial w}{\partial z}=0,$$

2 $$u\frac{\partial u}{\partial x}+\upsilon \frac{\partial u}{\partial y}+w\frac{\partial u}{\partial z}=\nu \frac{{\partial }^{2}u}{\partial {z}^{2}}-\nu {\prime} \frac{{\partial }^{4}u}{\partial {z}^{4}}-\frac{\sigma {B}_{0}^{2}}{\rho }u,$$

3 $$u\frac{\partial v}{\partial x}+\upsilon \frac{\partial \upsilon }{\partial y}+w\frac{\partial \upsilon }{\partial z}=\nu \frac{{\partial }^{2}\upsilon }{\partial {z}^{2}}-\nu {\prime} \frac{{\partial }^{4}\upsilon }{\partial {z}^{4}}-\frac{\sigma {B}_{0}^{2}}{\rho }\upsilon ,$$

4 $$u\frac{\partial T}{\partial x}+\upsilon \frac{\partial T}{\partial y}+w\frac{\partial T}{\partial z}=\frac{k}{\rho {C}_{p}}(\frac{{\partial }^{2}T}{\partial {z}^{2}})+\frac{2\mu }{\rho {C}_{p}}[{(\frac{\partial u}{\partial z})}^{2}+{(\frac{\partial \upsilon }{\partial z})}^{2}]+\frac{n}{\rho {C}_{p}}[{(\frac{{\partial }^{2}u}{\partial {z}^{2}})}^{2}+{(\frac{{\partial }^{2}\upsilon }{\partial {z}^{2}})}^{2}]+\frac{\sigma {B}_{0}^{2}}{\rho {C}_{p}}({u}^{2}+{v}^{2})+\tau [{D}_{B}\frac{\partial C}{\partial z}\frac{\partial T}{\partial z}+\frac{{D}_{T}}{{T}_{\infty }}{(\frac{\partial T}{\partial z})}^{2}],$$

5 $$u\frac{\partial C}{\partial x}+v\frac{\partial C}{\partial y}+w\frac{\partial C}{\partial z}={D}_{B}(\frac{{\partial }^{2}C}{\partial {z}^{2}})+\frac{{D}_{T}}{{T}_{\infty }}(\frac{{\partial }^{2}T}{\partial {z}^{2}}),$$

with allied the boundary conditions

6 $$\begin{array}{c}\begin{array}{ccc}u={U}_{w}(x,y)={U}_{0}{e}^{\tfrac{x+y}{L}}, & \upsilon ={V}_{w}(x,y)={V}_{0}{e}^{\tfrac{x+y}{L}}, & w=0,\end{array}\\ \begin{array}{ccc}k\frac{\partial T}{\partial z}=-{h}_{f}({T}_{w}-T), & {D}_{B}\frac{\partial C}{\partial z}+\frac{{D}_{T}}{{T}_{\infty }}\frac{\partial T}{\partial x}=0, & {\rm{at}}\,\,z=0,\end{array}\\ \begin{array}{lllll}u\to 0, & \upsilon \to 0, & C\to {C}_{\infty }, & T\to {T}_{\infty }, & {\rm{as}}\,\,z\to \infty .\end{array}\end{array}$$

Here, *U*_0_, *V*_0_, *v*, $\nu {\prime} =\tfrac{n}{\rho },\,{h}_{f},\rho ,\,A,\,\sigma$, *n*, *k*, *C*_*p*_, *D*_*B*_, *T*_*f*_, *L*, *τ*, and *D*_*T*_ are the constants, kinematic viscosity, couple stress viscosity, heat transfer coefficient, density, temperature exponent, electric charge density, couple stress viscosity parameter, thermal conductivity, specific heat, Brownian diffusion coefficient, convective fluid temperature below the moving surface, reference length, the quotient of the effective heat capacity of the fluid to the heat capacity of the nanoparticle material fluid, and thermophoretic diffusion coefficient respectively. The second term in equation 2 represents the couple stress fluid component along *x*− direction and third term relates the component of magnetic field along *x*− direction. Similarly, the second and third terms in equation 3 point out the couple stress fluid component and magnetic field component along *y*− direction. In equation 4, the second and third terms symbolize the viscous dissipation and the fourth term denote the Joule heating. The fifth term in equation 4 indicates the Brownian motion due to nanofluids and the second term in equation 5 designates the thermophoresis diffusion term due to nanofluids. Using the under mentioned transformations

7 $$\begin{array}{c}u={U}_{0}{e}^{\tfrac{x+y}{L}}f{\prime} (\eta ),\,\,v={U}_{0}{e}^{\tfrac{x+y}{L}}g{\prime} (\eta ),\,\,w=-\sqrt{\frac{v{U}_{0}}{2L}}{e}^{\tfrac{x+y}{2L}}(f+\eta \,f{\prime} +g+\eta g{\prime} ),\\ \eta =\sqrt{\frac{{U}_{0}}{2vL}}{e}^{\tfrac{x+y}{2L}}z,\,\,{T}_{w}={T}_{\infty }+{T}_{0}{e}^{\tfrac{A(x+y)}{2L}}\theta ,\,\,{C}_{w}={C}_{\infty }+{C}_{0}{e}^{\tfrac{A(x+y)}{2L}}\phi .\end{array}$$

Eq. (1) is satisfied inevitably and Eqs (2–6) take the form

8 $$f\prime\prime\prime -2(f{\prime} +g{\prime} )f{\prime} +(f+g)f{\prime\prime} -Kf{\prime\prime} {\prime\prime} -{M}^{2}f{\prime} =0,$$

9 $$g\prime\prime\prime -2(f{\prime} +g{\prime} )g{\prime} +(f+g)g{\prime\prime} -Kg{\prime\prime} {\prime\prime} -{M}^{2}g{\prime} =0,$$

10 $$\begin{array}{c}\theta {\prime\prime} -{\rm{\Pr }}\,A(f{\prime} +g{\prime} )\theta +{\rm{\Pr }}(f+g)\theta {\prime} +2{\rm{\Pr }}\,Ec({f}^{{\prime\prime} 2}+{g}^{{\prime\prime} 2})-K\,{\rm{\Pr }}\,Ec({f}^{{\prime\prime} 2}+{g}^{{\prime\prime} 2})\\ +{\rm{\Pr }}\,{M}^{2}Ec({f}^{{\prime} 2}+{g}^{{\prime} 2})+{\rm{\Pr }}\,Nb\theta {\prime} \varphi {\prime} +{\rm{\Pr }}\,Nt{\theta }^{{\prime} 2}=0,\end{array}$$

11 $$\varphi {\prime\prime} -ScA(f{\prime} +g{\prime} )\varphi +Sc(f+g)\varphi {\prime} +\frac{{N}_{t}}{{N}_{b}}\theta {\prime\prime} =0,$$

12 $$\begin{array}{c}\begin{array}{llllllll}f=0, & g=0,\, & f{\prime} =1, & g{\prime} =\alpha , & \theta {\prime} =-\gamma (1-\theta ), & Nb\varphi {\prime} +Nt\theta {\prime} =0 & {\rm{at}} & \eta =0,\end{array}\\ \begin{array}{lllll}f{\prime} (\infty )\to 0, & g{\prime} (\infty )\to 0, & \theta (\infty )\to 0, & \varphi (\infty )\to 0, & {\rm{as}}\,\,\eta \to \infty .\end{array}\end{array}$$

Here, prime denotes differentiation *w*.*r*.*tη*. However, *K*, *M*, *α*, Pr, *Sc*, *Ec*, *N*_*b*_, *γ* and *N*_*t*_ represent dimensionless couple stress parameter, Hartmann number, ratio of rates parameter, Prandtl number, Schmidt number, Eckert number, Brownian motion parameter, Biot number and thermophoresis parameter respectively. Values of these parameters are given as under:

13 $$\begin{array}{c}\begin{array}{llllll}K=\frac{v{\prime} a}{{v}^{2}}, & {\rm{\Pr }}=\frac{\mu {C}_{p}}{k}, & {\rm{Sc}}=\frac{v}{{D}_{B}}, & {M}^{2}=\frac{2\sigma {B}_{0}^{2}L}{\rho {U}_{w}}, & \gamma =\frac{h}{k}\sqrt{\frac{2\nu L}{{U}_{w}}}, & \alpha =\frac{{V}_{0}}{{U}_{0}},\end{array}\\ \begin{array}{ccc}Ec=\frac{{U}_{w}^{2}}{{C}_{p}({T}_{w}-{T}_{\infty })}, & {N}_{b}=\frac{\tau {D}_{B}}{\nu }({C}_{w}-{C}_{\infty }), & {N}_{t}=\frac{\tau {D}_{T}({T}_{w}-{T}_{\infty })}{v{T}_{\infty }}.\end{array}\end{array}$$

In non-dimension form, Skin friction coefficient and local Nusselt number are represented by

14 $$\begin{array}{ccc}{C}_{fx}{(\frac{{\mathrm{Re}}_{x}}{2})}^{\tfrac{1}{2}}={e}^{(\tfrac{3(x+y)}{2L})}f{\prime\prime} (0), & {C}_{fxy}{(\frac{{\mathrm{Re}}_{x}}{2})}^{\tfrac{1}{2}}={e}^{(\tfrac{3(x+y)}{2L})}g{\prime\prime} (0), & \frac{L}{x}N{u}_{x}{(\frac{{\mathrm{Re}}_{x}}{2})}^{\tfrac{1}{2}}=-\,{e}^{(\tfrac{(x+y)}{2L})}\theta {\prime} (0),\end{array}$$

where

15 $${\mathrm{Re}}_{x}={U}_{0}L/\nu $$

is the local Reynolds number.

## Series Solutions

To solve the presented modeled problem, Homotopy Analysis method (HAM) is engaged to obtain the series solutions for the system of nonlinear differential equations with allied boundary conditions. Over the years numerical techniques are developed but owing to obvious restrictions^42^, analytical techniques are adopted as an alternative by the scientists. Amongst these, Perturbation techniques are the most popular methods and are extensively applied in engineering and science problems^43^. One prime limitation of these techniques that they highly rely on small/large physical parameters, and owing to this deficiency these are valid only for weakly nonlinear problems and are not in the preferred list to solve the highly nonlinear problems. Thus, non-perturbation techniques like the variational iteration method^44^, the expansion method^45^, the Lyapunov’s artificial small parameter method^46^, and Adomian decomposition method^47^ and so on, are introduced to address this shortcoming of dependency on small/large parameters. But, the convergence of series solutions is not guaranteed in these methods. These are in principle applicable only for weakly nonlinear problems too. Whereas HAM suggested by Liao^48^ is a generalized analytical approach to address any system with strong nonlinearity, with ample choice to ensure series solutions’ convergence. This technique is even good for far-field boundary conditions in contrast with the numerical techniques. The basic features of this techniques are as under:

i. Perturbation technique only produces convergent solution when the parameter values are kept small or large but not for both cases. However, HAM solutions are independent of the choice of parameter’s values, as it generates solution on the idea of Homotopic deformation from an initial guess estimate to the final solution.
ii. The convergence is controlled using an additional parameter in the solution rather than using some physical parameter. This parameter does not have physical significance but its values help us to control the divergence of a solution. So a proper choice of the value of ℏ offers us a convergent solution^48^.
iii. HAM gives us the freedom to produce solutions in terms of polynomials, exponential, logarithmic or trigonometric functions by choosing a base function. By looking at the physical system we can define the base functions accordingly. Like if we have damping problem we can choose *e*^−*x*^ type base function, if we have some oscillating phenomenon, we can choose trigonometric functions etc.^48^.

A comprehensive detail of this method with examples may be found at^49^. The initial guesses with respective linear operators required for the particular problem are given as under:

16 $$\begin{array}{ll}{f}_{0}(\eta )=(1-{e}^{-\eta }), & {g}_{0}(\eta )=\alpha (1-{e}^{-\eta }),\\ {\theta }_{0}(\eta )=\frac{\gamma }{1+\gamma }{e}^{-\eta }, & {\phi }_{0}(\eta )=-\frac{\gamma }{(1+\gamma )}\frac{{N}_{t}}{{N}_{b}}{e}^{-\eta },\end{array}$$

17 $$\begin{array}{cccc}{{\bf{L}}}_{f}=f\prime\prime\prime -f{\prime} , & {{\bf{L}}}_{g}=g\prime\prime\prime -g{\prime} , & {{\bf{L}}}_{\theta }=\theta {\prime\prime} -\theta , & {{\bf{L}}}_{\varphi }=\varphi {\prime\prime} -\varphi ,\end{array}$$

with the following ensuing characteristics

18 $$\begin{array}{ll}{{\bf{L}}}_{f}({A}_{1}+{A}_{2}{e}^{\eta }+{A}_{3}{e}^{-\eta })=0, & {{\bf{L}}}_{g}({A}_{4}+{A}_{5}{e}^{\eta }+{A}_{6}{e}^{-\eta })=0,\\ {{\bf{L}}}_{\theta }({A}_{7}{e}^{\eta }+{A}_{8}{e}^{-\eta })=0, & {{\bf{L}}}_{\phi }({A}_{9}{e}^{\eta }+{A}_{10}{e}^{-\eta })=0,\end{array}$$

in which *A*_*i*_(*i* = 1 − 10) are the arbitrary constants.

## Convergence Analysis

**Figure 2:**
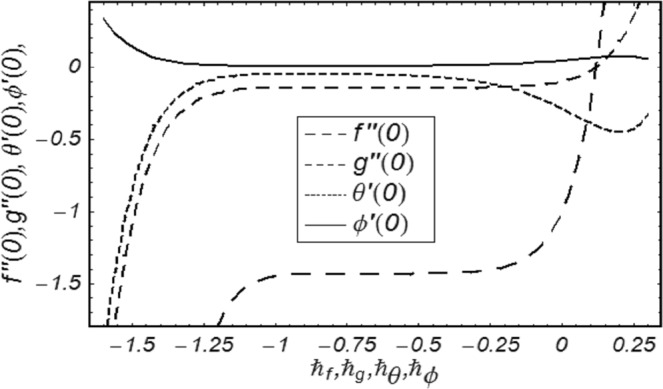
*ℏ*− curves for *f*, *g*, *θ*, *ϕ*.

In this section we will define the series solutions’ convergence by via HAM. The HAM solutions encompass auxiliary parameters *ℏ*_*f*_, *ℏ*_*g*_, *ℏ*_*θ*_ and *ℏ*_*ϕ*_. These parameters play a title role in controlling and regulating the convergence regions of resultant series solutions. To have the values of same parameters, *ℏ*− curves are obtained at 9^*th*^ order of approximation. Figure 1 shows the boundaries of the convergence regions −0.9 ≤ *ℏ*_*f*_ ≤ −0.5, −1 ≤ *ℏ*_*g*_ ≤ −0.4, −1.0 ≤ *ℏ*_*θ*_ ≤ −0.5 and −1.15 ≤ *ℏ*_*ϕ*_ ≤ −0.5. Table 1 presents the numerically calculated values of convergence up to 25^*th*^ order of approximations and it can also be presented as the counter check to *ℏ*− curves drawn in Fig. 2. Both Fig. 2 and Table 1 are correlated and are in good concurrence.

**Table 1:** Homotopy series solutions’ convergence for various order of estimations.

Order of estimation	−*f*″(0)	−*g*″(0)	−*θ*′(0)	−*ϕ*′(0)
1	1.4013	0.14013	0.196030	0.0032671
10	1.4357	0.14357	0.050620	0.0084366
15	1.4359	0.14359	0.048845	0.0081408
20	1.4359	0.14359	0.048492	0.0080819
25	1.4360	0.14360	0.048409	0.0080682
30	1.4360	0.14360	0.048409	0.0080682

## Discussion

The goal of this portion is to portray the significant characteristics of arising parameters on velocity components, temperature, and concentration distributions, Skin friction and the Nusselt number.

**Figure 3:**
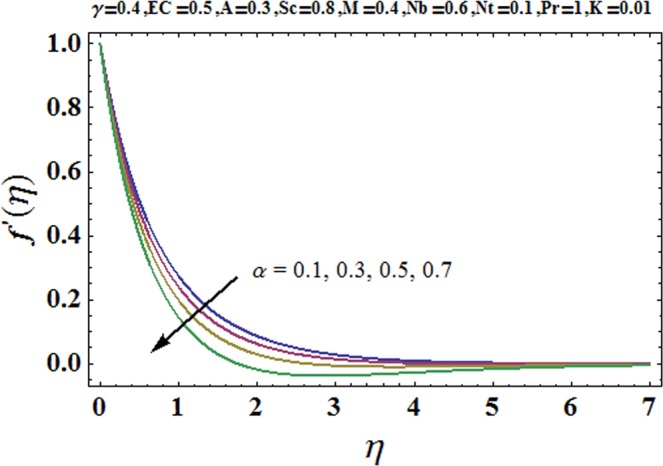
Variation of *α* versus *f*′(*η*).

**Figure 4:**
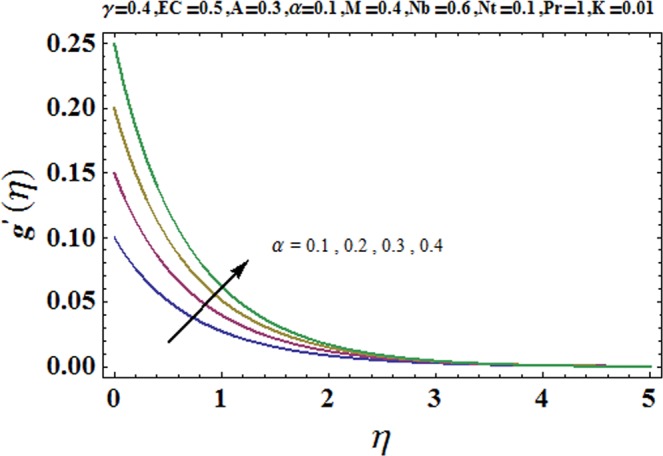
Variation of *α* versus *g*′(*η*).

**Figure 5:**
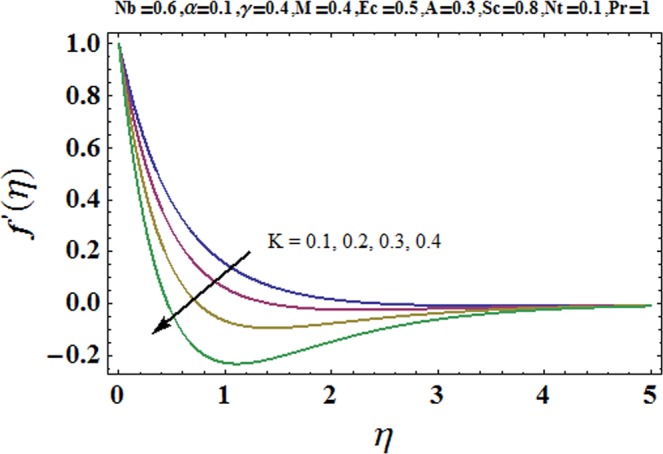
Variation of *K* versus *f*′(*η*).

**Figure 6:**
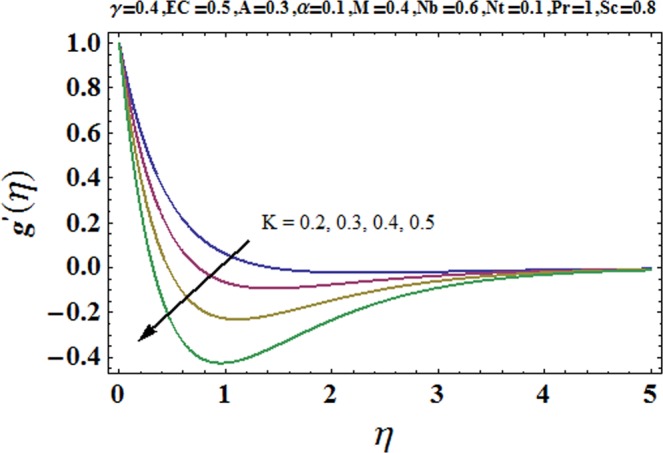
Variation of *K* versus *g*′(*η*).

**Figure 7:**
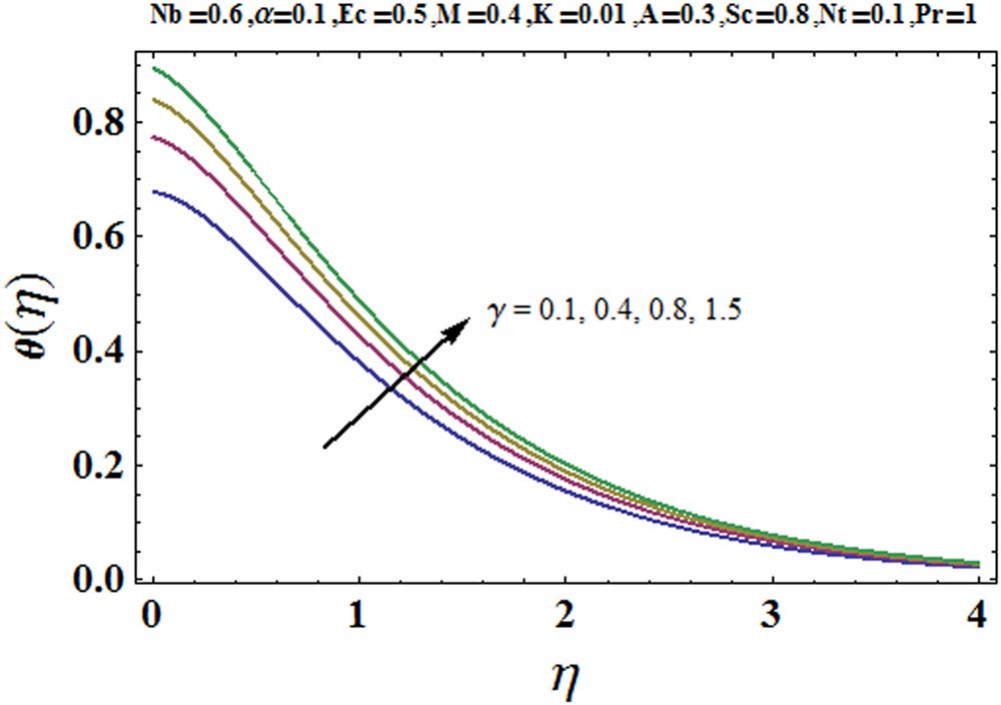
Variation of *γ* versus *θ*(*η*).

**Figure 8:**
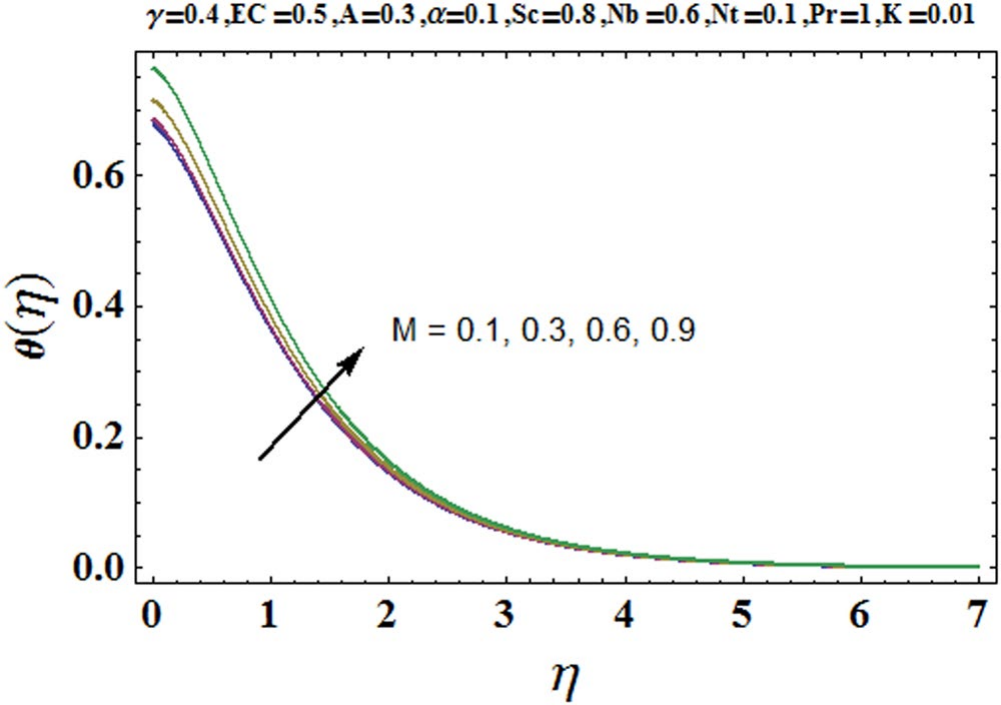
Variation of *M* versus *θ*(*η*).

**Figure 9:**
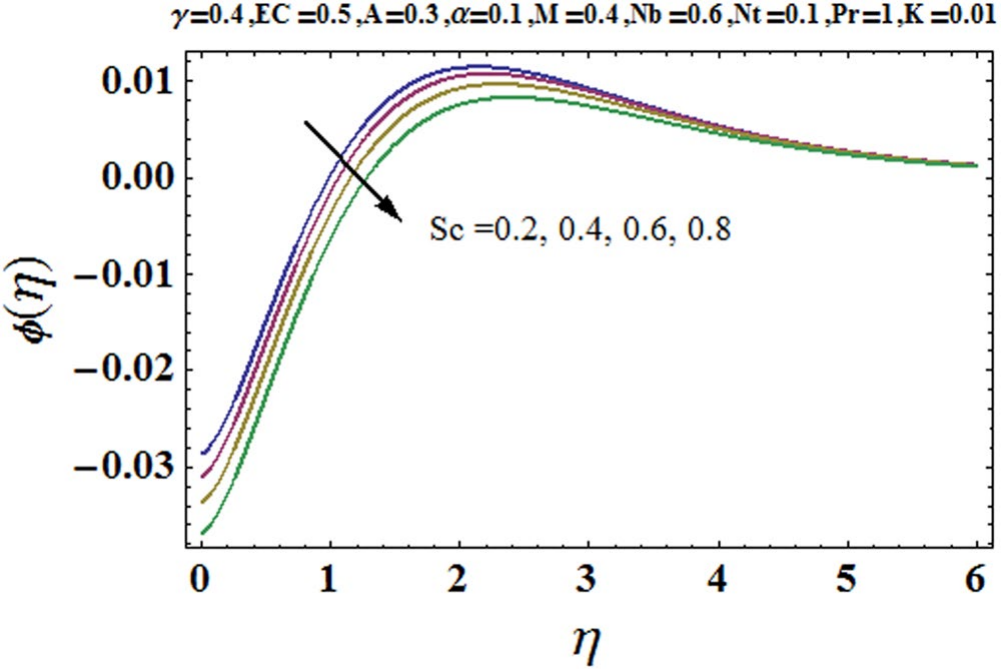
Variation of *Sc* versus *ϕ*(*η*).

**Figure 10:**
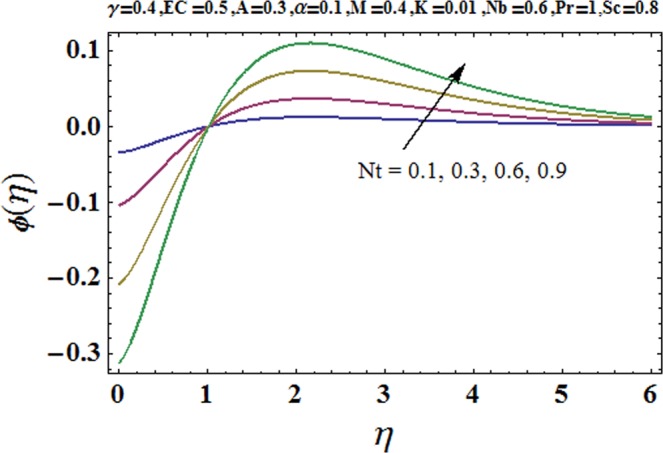
Variation of *Nt* versus *ϕ*(*η*).

**Figure 11:**
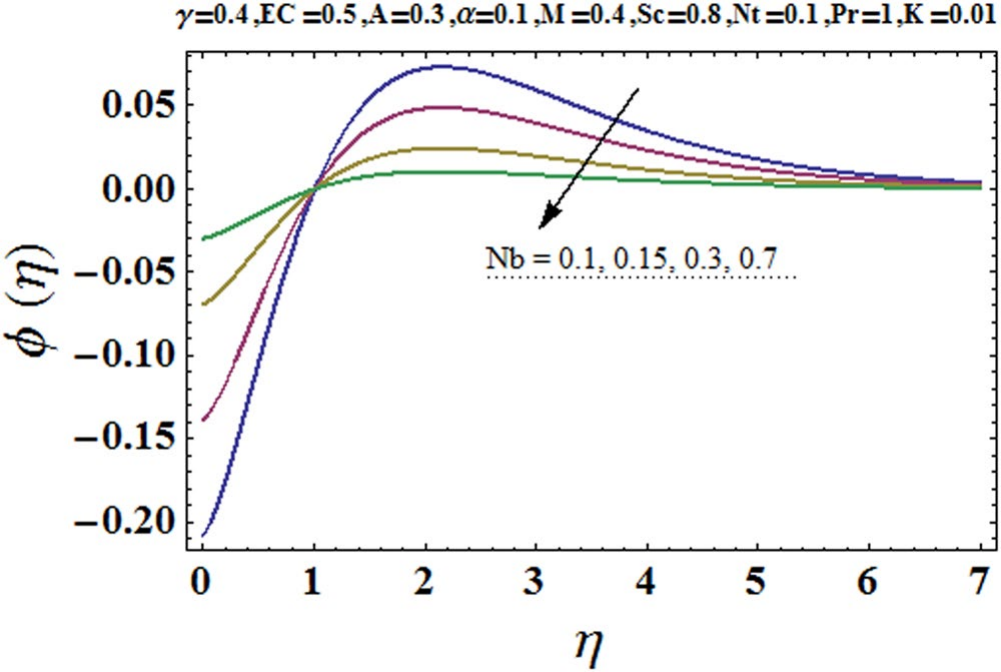
Variation of *Nb* versus *ϕ*(*η*).

**Figure 12:**
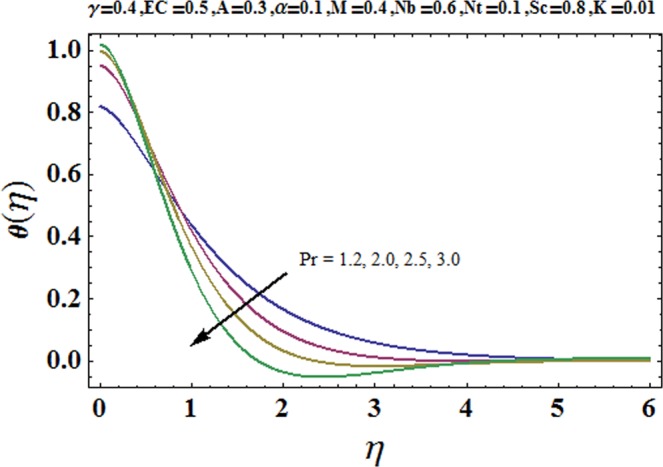
Variation of Pr versus *θ*(*η*).

**Figure 13:**
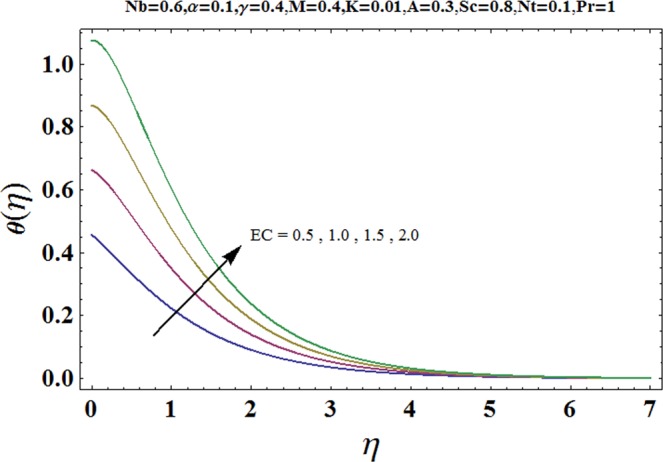
Variation of *Ec* versus *θ*(*η*).

**Figure 14:**
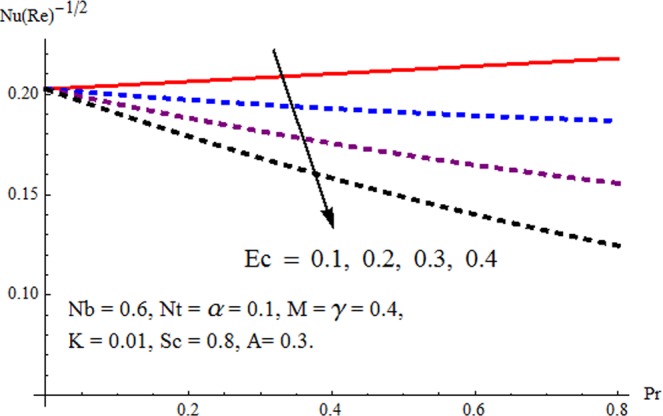
Variation of Pr and *Ec* versus −*θ*/(*η*).

**Figure 15:**
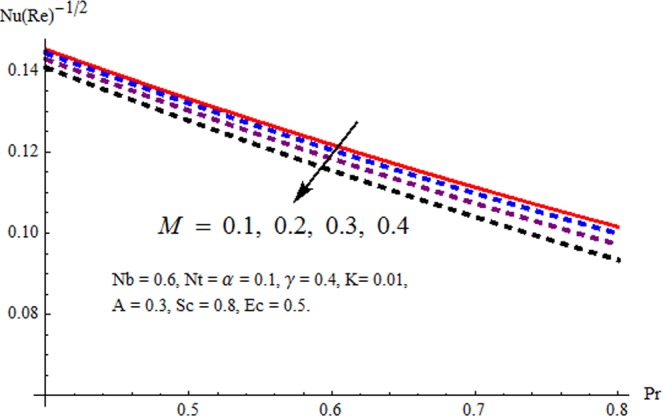
Variation of Pr and *M* versus −*θ*/(*η*).

Figures 3 and 4 are drawn to portray the consequence of the ratio of rates parameter *α* on velocity components *f*′ and *g*′ along *x*− and *y*− axes respectively. It is noticed that an escalation in the values of *α*, *f*′ decreases however *g*′ shows an opposite behavior. This is because of the fact that the rate constant of velocity component along the *y*− axis is more dominant in comparison to the velocity component along the *x*− axis. Figures 5 and 6 elucidate the impact of couple stress parameter *K* on the velocity components *f*′ and *g*′ respectively. Both velocity components decrease with rise in the values of *K*. The values of *K* are directly linked with couple stress viscosity parameter *n*. Higher values of *K* means more viscosity which hinders the movement of the fluid and finally decrement in the velocity components is witnessed. Figure 7 is plotted to show an impact of the Biot number *γ* on temperature profile. From the curves of *γ*, it is revealed that temperature distribution upsurges for incremented values of *γ*. Actually, escalated heat transfer coefficient is perceived for growing estimates of *γ* which eventually rises the temperature of the fluid. It is also comprehended that temperature of the fluid rises more rapidly near the stretched surface for incremented values of *γ*. The effect of Hartmann number *M* on the temperature field is showed in Fig. 8. It is comprehended that the temperature profile upsurges for higher values of *M*. Augmentation in the Lorentz force is observed because of augmented values of *M*; due to this phenomenon, resistance in the fluid motion is experienced which results in more collisions of molecules in the fluid and eventually upsurge in fluid’s temperature is witnessed. Figure 9 demonstrates the impact of Schmidt number *Sc* on the concentration distribution. A feeble mass diffusivity is witnessed for large values of *Sc*. This weak mass diffusivity will affect the mass concentration of the fluid and consequently decrease in the concentration field is witnessed. Figure 10 is illustrated to depict the behavior of the thermophoresis parameter *Nt* on concentration distribution. Mounting values of *Nt* push nanoparticles far away from the warm surface, which results in an enriched concentration distribution. In Fig. 11, the effect of Brownian motion parameter *Nb* on concentration field is portrayed. For higher values of *Nb*, reduction in concentration profile is witnessed. Actually, mounting values of *Nb* are the root cause to boost the random motion amongst nanoparticles and as a result decrease in concentration of the fluid is witnessed. The impact of Prandtl number Pr on the temperature field is depicted in Fig. 12. As Pr is directly proportional to momentum diffusivity and inversely proportional to thermal diffusivity. Higher values of Pr means there is strong momentum diffusivity as compared to the thermal diffusivity and such weak thermal diffusivity relates to the weaker temperature profile. The effect of Eckert number *Ec* on the temperature distribution is illustrated in Fig. 13. From figure, it is noted that temperature field is an escalating function of *Ec*. This is because of frictional drag that becomes the main source to raise heat energy in the fluid. Figures 14 and 15 are sketched to show the impact of the Hartmann number *M*, the Eckert number *Ec* and the Prandtl number Pr on Nusselt number −*θ*′(0). It is detected that Nusselt number is decreasing function of all three parameters. Nusselt number is a non-dimensional number that is used to gauge the heat transfer between a solid body and a moving fluid. The reason for decrease in value of Nusselt number is because of decline in the natural convection of nanofluids. Actually, the augmented Lorentz force due to the strong magnetic field weakens the natural convection for nanofluids.

Tables 2 and 3 portray the Skin friction coefficients along *x*− and *y*− axes for varied values of ratio parameter *α*, couple stress parameter *K* and Hartmann number *M* and It is gathered that the Skin friction coefficients are enhanced for increasing values of *α*, *K* and *M*. Table 4 is constructed for Nusselt number versus varied values of involved parameters. It is witnessed that Nusselt number upsurges for *γ*, *α*, *A* and shows decreasing tendency for growing values of *K*, *M*, Pr, *Ec* and *Sc*. Table 5 is initiated to depict comparison of the presented problem in limiting case to a previously done exploration^50^ and all obtained results are found in an tremendous concurrence. Table 6 is erected with the same objective to authenticate our obtained results with Hayat *et al*.^51^ in limiting case. An excellent concurrence is achieved when the results are compared.

**Table 2:** Estimates of Skin friction coefficient $({C}_{f}{{\rm{Re}}}_{x}^{1/2})$ for *α*, *K* and *M*.

α	K	M	$-\,{{\boldsymbol{C}}}_{{\boldsymbol{f}}}{\bf{R}}{{\bf{e}}}_{{\boldsymbol{x}}}^{1{\boldsymbol{/}}2}$
0.1			1.43588
0.2			1.45336
0.3			1.51480
0.1	0.02		1.48194
	0.03		1.58900
	0.01	0.1	1.37723
		0.2	1.38939
		0.3	1.40891

**Table 3:** Estimate of Skin friction coefficient $({C}_{g}{{\rm{Re}}}_{x}^{1/2})$ for *α*, *K* and *M*.

α	K	M	$-{C}_{g}{{\rm{Re}}}_{x}^{1/2}$
0.1			0.143578
0.2			0.299583
0.3			0.467421
0.4			0.646402
0.1	0.02		0.147835
	0.03		0.154193
	0.04		0.164643
	0.01	0.5	0.146938
		0.6	0.150971
		0.7	0.155608

**Table 4:** Numerically calculated values of local Nusselt number for $\gamma ,\,\alpha ,\,K,\,M,\,\Pr ,\,Ec,\,A$ and *Sc*. when *Nb* = 0.6, *Nt* = 0.2.

γ	α	K	M	Pr	Ec	A	Sc	${\rm{Nu}}/R{e}_{x}^{1/2}$
0.2	0.3	0.02	0.3	1.0	0.1	0.2	1.0	0.1302
0.5								0.2450
0.7								0.2945
0.9								0.3315
0.2	0.5							0.1314
	0.7							0.1327
	0.8							0.1330
	0.3	0.03						0.1286
		0.04						0.1225
		0.05						0.1045
		0.01	0.3					0.1301
			0.5					0.1270
			0.7					0.1170
			0.1	1.2				0.1310
				1.3				0.1305
				1.4				0.1295
				1.0	0.2			0.1082
					0.3			0.1045
					0.4			0.1011
					0.1	0.3		0.1092
						0.4		0.1138
						0.5		0.1179
						0.1	0.4	0.1310
							0.5	0.1306
							0.7	0.1302

**Table 5:** Comparison table erected for wall temperature gradient *θ*′(0) in limiting case in absence of *α*, *γ*, *K*, *M* and nanofluid with Liu *et al*.^50^ and Magyari. & Keller^52^.

Pr	A	*θ*′(0)	*θ*′(0)	*θ*′(0)
		^52^	^50^	Present
1	−1.5	0.377413	0.37741256	0.37741301
	0	−0.549643	−0.54964375	−0.54964339
	1	−0.954782	−0.95478270	−0.95478277
	3	−1.560294	−1.56029540	−1.56029499
5	−1.5	1.353240	1.35324050	1.35324055
	0	−1.521243	−1.52123900	−1.52123893
	1	−2.500135	−2.50013157	−2.500135210
	3	−3.886555	−3.88655510	−3.88655512
10	−1.5	2.200000	−2.20002816	2.20000798
	0	−2.2574249	−2.25742372	−2.25742910
	1	−3.660379	−3.66037218	−3.66037911
	3	−5.635369	−5.62819631	−5.635316812

**Table 6:** Comparison table erected for −*f*″(0), −*g*″(0) and *f*(∞) + *g*(∞) in limiting case in absence of *K*, *M* and nanofluid with Hayat *et al*.^51^.

*α*	Hayat *et al*.^51^			Present		
	−*f*″(0)	−*g*″(0)	*f*(∞) + *g*(∞)	−*f*″(0)	−*g*″(0)	*f*(∞) + *g*(∞)
0	1.281809	0	0.905644	**1.281809**	**0**	**0.905644**
0.5	1.569889	0.784944	1.109182	**1.569889**	**0.784944**	**1.109182**
1.0	1.812751	1.812751	1.28077	**1.812751**	**1.812751**	**1.28077**

## Concluding Remarks

The flow of couple stress nanofluid past an exponential stretched surface with effects of magneto hydrodynamic, viscous dissipation and Joule heating is examined here analytically. Impacts of convective heat and zero mass flux conditions are also deliberated. The system of nonlinear differential equations is solved using Homotopy analysis method. Influences of various parameters on velocity field, temperature field, concentration distribution, Skin friction coefficient and the local Nusselt number are depicted through graphical illustrations. The significant findings of the present problem are summarized as follows:

- For growing values of Brownian motion and thermophoresis parameters, the nanoparticle concentration distribution shows decreasing and increasing behavior respectively.
- Effective enhancement of ratio of rates parameter *α* accounts for decrease in velocity component *f*′ and increase in the other velocity component *g*′.
- The temperature distribution enhances versus increasing values of Biot number and Hartmann number.
- For the values of *K*, *M*, Pr, *Ec* and *Sc*, Nusselt number shows escalating behavior.
